# All you need is [somebody’s] love “third-party reproduction” and the existential density of biological affinity

**DOI:** 10.1007/s40592-024-00212-3

**Published:** 2024-11-15

**Authors:** Diogo Morais Sarmento Madureira

**Affiliations:** Institute for Political Studies, Catholic University of Portugal, Lisboa, 1300 Portugal

**Keywords:** Natural bonds, Third-party reproduction, Filiation, Children’s rights, Family policies, Kinship, Biological family, Father’s love, Human affinity, Personality, Right to a child

## Abstract

What is the true significance of biological kinship? During the last decades, it seemed to be uncontroversial that abandoned and even adopted people feel the negative impact of biological parents’ absence throughout life in several ways (Miller et al. 2000; Keyes, Margaret A., Anu Sharma, Irene J Elkins, and William G. Iacono, Matt McGue. 2008. The Mental Health of US Adolescents Adopted in Infancy. *Archive Pediatric Adolescense Medicine* 162(5): 419–425.). However, in the case of people conceived via “third-party reproduction”, especially in sperm donation, the disruption of the kinship network derived from natural bonds tends to be presented as something irrelevant. This article disputes that assumption, explores its relationship with a deconstructivist vision that presents kinship as a purely social construct and defends the personal and existential value of a person’s biological bonds with her parents. While analysing the anthropological shift inherent to the way some political discourses present the nuclear family and heterologous biotechnology, it proposes renewed philosophical attention on the significance of filiation and human affinity. This article argues for the density of genealogical ties and defends that the consecration of an individual “right to a child”, namely (but not exclusively) through the normalised access to sperm banks, is incompatible with the rights of the child, since it deprives people from knowing not only who but also *how* is their father.

## Introduction

The presentation of sperm donation as something positive and altruistic, which exists to alleviate the suffering of people who cannot have children due to a disease or not having a relationship with a man, has strongly influenced political decisions in the last few years (J. Laing and Oderberg 2005). In Portugal, for example, this perspective motivated the replacement of the previous 2006 law on assisted reproductive technologies (ART) with the current one, from 2016 (Assembleia da República 2016). Donor anonymity was one of the clauses that ended up being declared unconstitutional in this new law from 2016 whose main difference to the previous law is that this one faced access to sperm banks as a remedy for a health problem, while the new one extends access to every woman, hence converting it from a subsidiary into an alternative method of reproduction (Tribunal Constitucional 2018)^1^. By doing so, the a priori separation between fathers and children, previously admitted only as an extreme solution for extreme cases, became accepted as the legitimate result of an individual choice, with no need for any other justification besides the desire to have a child. Such separation takes place through the legitimation of agreements essentially consisting of the following: a woman and a man decide to generate a person whom only the woman intends to raise and take care of (alone or together with another individual); the man, for his part, only accepts to generate that person precisely on the condition that he does not have to raise her. In most cases, indeed, the objective is that the man remains anonymous since most women resorting to sperm insemination do not want to connect with the donor (Beeson et al. 2011).

As many of the approximately 6.5 million people conceived via third-party reproduction (TPR) worldwide (Zillén, Garland, and Slokenberga 2017) started raising their voices against donor anonymity, both scientific research and political decisions converge with what is stipulated, for example, in article 8 of the European Convention on Human Rights: the right to know mother and father is a fundamental human right. Despite some ambiguous readings on TPR, the European Court of Human Rights defended the individual’s vital interest in obtaining information about crucial elements of personal identity, such as knowing who are one’s parents (European Court of Human Rights 2003; 2006; 2011b; 2011a; 2013). Sweden in 1984, Germany in 1989, and the United Kingdom in 2005, among others (Norway or Switzerland, for example), have also eradicated donor anonymity (Blyth et al. 2012), thus echoing voices like Joanna Rose’s, the “donor-conceived” woman responsible for the legal case that ended UK’s anonymity law (Great Britain. England and Wales. Supreme Court of Judicature, High Court of Justice 2002).

Anonymity is a relevant ethical issue in this discussion, since among other consequences, it leads, for example, to stark discrimination: while adopted children benefit from a broad social and scientific consensus on the need to know their origins, children conceived via sperm banks are forbidden to do so in countries where anonymity is still permitted, such as the US or Spain. However, as Joanna Rose explains in the exhaustive reflection she undertook on the way literature, politics, and social representation neglect “the personal and social effects of disrupting the unity of biological and social relatedness for the offspring” (Rose 2009), there are other ethical problems to be taken into account. Instead of being considered something to dissuade as a general rule, disconnecting the biological and social components of kinship tends to appear as something irrelevant in the context of TPR. An essential dimension of the right to personhood and identity is therefore neglected.

To understand this situation it is extremely useful to acknowledge the influence of two close ideas: one is that “affections are construed; they are not in the blood”; the other is well resumed in the colloquial sentence “all you need is love”, an attractive and *quasi* intuitive aphorism which in the context of this debate shall notwithstanding be understood as if it was indifferent or irrelevant to have the biological parents’ love — to put it simply: if you are loved it doesn’t matter who loves you. These ideas connect, in turn, with two other strongly related convictions. One says that just like masculine and feminine, fatherhood and motherhood are “performative” categories that shall be deconstructed, meaning that they constitute social roles that can be reinterpreted by other people than biological parents (Levine 2008; Robaldo 2011). The other departs from the same deconstructivist epistemology to affirm that it is law — not biology or nature — that determines father and mother; hence the donor shall not be considered father (Anne Cadoret 2003; Bruton 2018).

The perspective just described characterizes what can be denominated as a deconstructivist vision. This article critically analyzes it, along with its respective premises and arguments, through complementary and interconnected counterarguments that could be condensed into two main ideas: (1) biology is biographical; (2) love is not importable, i.e., it is not alienable, because the responsibility to love cannot be transferred (Moschella 2017).

To develop these ideas we address scientific evidence and argue that TPR implies the deliberate alienation of kinship duties and rights by voluntarily breaking the inherent bonds derived from the connection between the conceived person, their biological parents and the whole network of familiar affinities (grandparents, brothers and sisters, aunts, cousins). It is true that, regardless of whether such agreements materialize through sexual intercourse or biotechnology, a demo-liberal State cannot stop them from occurring, but the question is whether that State, which is fundamentally committed to the protection of the weakest and human dignity, should deter those agreements or not. Forbidding the practice does not seem to be a solution, but this article argues that the State must recognize the right of the child to an ongoing connection with the biological mother or father and ensure that child the possibility of knowing her biological parents in the proper sense of the word, i.e., not only to know *who* they are but — equally important — *how* they are. Our main argument is in line with that of James Vellemanm (Velleman 2005, 2008a, b).

It is logical that among healthy women, those interested in sperm donation are mainly women unrelated to a man (Beeson et al. 2011; VARTA 2022), which means that converting TPR from a subsidiary into an alternative means of reproduction results, in practice, in the absence of a father in the life of the person thus generated (Blyth 1999; Vanfraussen et al. 2002; Kirkman 2003; Cahn and Cahn 2008). By expanding the access of all women to sperm banks, just as the cited Portuguese law and similar ones worldwide do, the State accepts as valid the thesis of the irrelevance not only of the parents’ sex but also of the parents’ kinship, thus authorizing life projects which have no intention to provide the person to be born the company of even an adoptive father. Indeed, such laws accept as truth the idea that it is possible to reduce parents to donors and that they are “third parties” for the children. The impossibility of cultivating biological ties through an affective relationship with parents also means the impossibility of feeding any affinity with half of a person’s family. This makes it harder for people generated via TPR “to connect with one’s roots; to complete one’s life (hi-) story; to understand where one’s traits come from; to discover one’s defining characteristics and capabilities; and to map out one’s ancestral history” (Ravelingien et al. 2013, p. 257).

## Biology is biographic

In 2018, within the scope of the Bioethics *États Généraux* called by the French government to prepare new legislation and which was dominated by the possibility of coming into force in France a TPR law similar to the Portuguese one, Aude Mirkovic, spokesman for the French association “Jurists for Childhood”, published a book with this sharp introductory summary:

The PMA for single women and women’s unions is one of the flagship measures of the revision of the 2011 bioethics law and the public’s attention on the matter is strong, but when it comes to facing this non-therapeutic PMA, society must choose: where do we want to go with artificial reproduction techniques? Should PMA be an exceptional measure, designed to compensate for a medical problem, or become a habitual procreation mode that drags society into a new anthropology, to use the National Ethical Advisory Council’s terms? The French who say they are in favour of WFP for single women and women’s unions would have the same opinion if the question were asked from the child’s point of view: ‘Do you think that the law should organise the conception of children deliberately and legally deprived of father’? (Mirkovic 2018).

The erasure of the father, or the normalization of its absence from his biological children’s lives, is a direct consequence of the deconstructivist dynamic generated by the convictions “affections are construed; they are not in the blood” and “all you need is love”. In this first point, we address two arguments that strongly contribute to that outcome.

First, the *argument of the nonconclusive information*, according to which existing empirical research is not clear on the harms of cutting the connection with the biological father and, concretely, of sperm donation.

Intimately associated with this argument there is a second one, known in bioethics as the *argument of existential debt*, that consists of the following reasoning: despite the harm that the child may experience for lacking a father connection, she will still be glad to be alive; would she prefer not to be born rather than be born but not know her biological father?

The underlying conclusion to both arguments is, on the one side, that those who oppose TPR shall be the ones who must prove its harms. On the other side, even though they could prove it, the practice would still be equally morally justified, because the harm of not having one’s biological father would never be so great that people would prefer not to be born — bringing someone into the world inevitably causes them to suffer and if a parent believes that the goods of life outweigh the harms, then it is not wrong to procreate not even via confidential sperm donation.

Our response to each of these arguments aims to show, among other things, that they jointly ignore a very important principle in bioethics, especially in the context of public policies related to reproductive biotechnology, which is recalled by the sound teaching that not everything technically possible is ethically acceptable: the principle of prudence or precaution. Aude Mirkovic accurately resumes this point:

The Convention on the Rights of the Child proclaims for all of them the right to know and be raised by their parents whenever possible. Now, a procreation technique that deliberately and definitively erases the father is not compatible with such a right. If we think that 2018 children do not need a father, we need to start by proving it, because experience invites us to think otherwise; afterwards, we will need to correct CRC (…) It would perhaps be preferable to accept that, in a rule of law State, the satisfaction of the desires of some finds its limit in the respect for the rights of others and includes children. (Mirkovic 2018).

### The argument of non-conclusive information

Several authors have argued that the biographical weight and significance of the biological connection includes more than just data on who is one’s father (Callahan 1992; Velleman 2005; Laing 2006; Guichon et al. 2012; Moschella 2014). But is that position empirically grounded? A good way to introduce the response could be by citing a 2011 report entitled “Men in Families”, from the UN Economic and Social Council:

Regardless of the mechanisms and effects that a parent produces, their presence or involvement is associated with several benefits for children, both in the short term and over time (UN Department of Economic and Social Affairs - Division for Social Policy and Development 2011, 58).

A 2007 UNICEF report defended similar conclusions (UNICEF Innocenti Research Centre 2007, p. 23). Another study cited at a UN expert meeting titled “Family Policies and the 2030 Sustainable Development Agenda states that “there is copious research supporting the notion that involved fatherhood is crucial for the development of healthy, well-functioning families” (Behson and Robbins 2016, 1), a conclusion corroborated by other authors (Hofferth 2006; Cabrera and Catherine S. Tamis-LeMonda 2013). The trivialisation of the separation between children and biological father through TPR is at odds with the conclusion that “the continuity of biological, emotional and legal paternity and motherhood constitutes an advantage for the child”, as affirmed in a European Parliament resolution from 1989 (Parlamento Europeu 1989, 46). It also seems to violate the Convention on the Rights of the Child, which stipulates the children’s fundamental right to know their parents and to preserve the union between them as a general rule — *vide*, for example, § 7 to § 9 (ONU 1989). That right shall not be subordinated to “parental rights or interests”, contrarily to what authors like Sally Haslanger defend (Haslanger 2009) and according, for example, to the National Council of Ethics for Life Sciences of Portugal, which regarding TPR techniques stated that “due to its nature and vulnerability, the being that will be born is the one who lacks most protection” (Conselho Nacional de Ética para as Ciências da Vida 2016), an understanding that also prevailed in the UK, at least until recently (McWhinnie 2001, p. 808).

The scientific knowledge accumulated by research on biological and non-biological families over decades in which the issue was not as politically controversial as it is today, offered empirical support for the described normative heritage (Schechter 1964; Amato 1994; Cherlin et al. 1998; Flinn, Leone, and Quinlan 1999). Indeed, “Several decades of research linked privacy of the father in childhood to higher risks of depression and other forms of psychopathology” (Tyrka et al. 2008, 1147), and rather than sustaining the thesis of the irrelevance of the father’s absence, multidisciplinary scientific evidence corroborates the ideal of growing up with one own’s mother and father:

It is not simply the presence of two parents but the presence of two biological parents that seems to support children’s development. (…) [R]esearch demonstrates that family structure matters for children and the family structure that helps children the most is a family headed by two biological parents in a low-conflict marriage. Children in single-parent families, children born to unmarried mothers, and children in stepfamilies or cohabiting relationships face higher risks of poor outcomes than do children in intact families headed by two biological parents.” (Moore, Jekielek, and Emig 2002, 1).

Many other studies defend the same conclusion (Phares and Compas 1992; Amato 1998, 2012; Popenoe 1999; Regnerus 2012), therefore converging with Sarah McLanahan’s analysis of the work her teams have conducted over the years:

If we were asked to design a system for making sure that children’s basic needs were met, we would probably come up with something quite similar to the two-parent ideal. The fact that both parents have a biological connection to the child would increase the likelihood that the parents would identify with the child and be willing to sacrifice for that child, and it would reduce the likelihood that either parent would abuse the child (S. McLanahan and G.Sandefur 1994, 38).

McLanahan corroborated this conclusion in 2005 (McLanahan et al. 2005), as well as Mary Parke (Mary Parke 2003), Wendy Manning and Michael Lamb: “The advantage of marriage appears to exist primarily when the child is the biological offspring of both parents.” (Manning and Lamb 2003, p. 890). The research institution Child Trends synthesizes the evidence in the following way:

Among young children, those living with no biological parents or in single-parent households are less likely than children with two biological parents to exhibit behavioural self‐control, and more likely to be exposed to high levels of aggravated parenting, than are children living with two biological parents. (…) Among children in two‐parent families, those living with both biological parents in a low‐conflict marriage tend to do better on a host of outcomes than those living in step-parent families. Outcomes for children in stepparent families are in many cases similar to those for children growing up in single‐parent families. (Child Trends 2014, 2).

The disproportionate way in which people raised without one or both biological parents face diverse kinds of problems^2^ by comparison to those who benefit from the coexistence with both (Paquette 2004; Amato 2011; Potter 2012; Child Trends 2014) is acknowledged even by authors from contrasting political perspectives who recognize that, as a rule, the absence of one or both biological parents can consistently be regarded as a strong predictor of emotional, psychological and behavioural problems during infancy and adolescence (Reimann 1997; Manning and Lamb 2003; Lamb 2010; Rosenfeld 2010). Therefore, existing evidence comparing the results of biological and non-biological children in a wide variety of indicators confirms that “genealogical bewilderment” matters and there are indeed strong reasons for seeing the biogenetic family as the “gold standard” (Sants 1964; Winkler and Mitford 1986; McWhinnie 1986). In other words, it supports the argument that despite multiple cases of people growing up normally without their natural parents’ presence, the separation between them cannot work as an ideal for public policies, because it cannot be considered a good option not even when it is the better option, as happens in extreme circumstances. This is corroborated by scientific research on adopted children (Smit 2002; Juffer and van IJzendoorn 2005; Keyes et al. 2008; van den Dries et al. 2009; Fullerton 2016), as well as by complementary evidence demonstrating the disproportional disadvantages of “donor-conceived” people when compared with peers raised by their biological parents (McWhinnie 1998, 2006; Allen 2013; Persaud et al. 2017; Martin 2017). In addition to all the authors already mentioned, one could also cite Donald Sullins, who has been carrying out a detailed critical analysis of previous studies and making his assessment of the USA’s national longitudinal data since at least 2015 (Sullins 2015a, b, 2016, 2021):

An extensive and diverse body of research finds that, compared to children continuously living with two parents, married parents, or their biological parents, children in other family arrangements consistently experience lower emotional well-being, physical health and academic achievement (…) This evidence [from Sullins’ 2021*study] strongly supports the claim that maximum child development occurs only in the persistent care of both of the child’s biological parents.* (Sullins 2021)

Apparently, all this body of research is unknown even to authors like Daniel Groll (Groll 2021), who recognizes some relevance to biological ties and is openly against anonymity in TPR practices, but is especially ignored by authors who deny and undervalue the biographical significance of biological connection for people born from TPR (Witt 2005; Leighton 2012; de Melo-Martín 2014).

### The argument for the existential debt

Within the context of the present discussion, this argument is frequently presented through a rhetorical question (ex: would “donor-conceived” people prefer to know their father or not to be born at all? ), or through categorical sentences like that of the once-president of UK’s Human Fertilisation & Embryology Authority, Emily Jackson: “existence must always be judged preferable to non-existence” (Rose 2009, p. 250).^3^

The existential debt argument has a direct link with Derek Parfit’s *non-identity problem* (Parfit 1986). As he explains through the depletion case, a procreation act may cause harm to the future person, but that person who exists and suffers as an effect of that act would never have existed without it, so she must be thankful for that act, which is not wrong. A different perspective is developed here and aligns with that of authors like James Velleman or James Woodward (Woodward 1986; Velleman 2008b). In the particular case of donor insemination, what makes the act under scrutiny wrong is the intentional and unjustifiable violation of the future person’s right against being brought into an existence without father.

If a human being has already been generated and exists as a fetus, the ethical appreciation of the decision to allow him to be born — even knowing that will be deprived of the father’s company — may be limited by the concrete existence of that human being. However, if we are not dealing with the concrete existence of anything (not even a cell or an embryo), then we are not harming anyone by defending that there is no reason to restrict to only two options the horizon of possibilities opened up to the agent’s decision: (a) having a child without a father; (b) not having the child. Since, in principle, everybody has a mother and a father, a third possibility has necessarily to be admitted: having a child and giving her her mother and father. Society, in the person of the legislator, must formulate its ethical judgment by reference to this third option and not ignore it a priori, because it harms nobody and fully respects the simultaneously diverse and exclusive nature of the reproductive relationship — the very same nature that structures the original meaning of the terms “mother” and “father”.

The existential debt argument often promotes a pernicious tendency to confuse the critic with the banalization of TPR (thus converted into an alternative means of procreation) with an attack on the dignity of people born from it. However, there is nothing wrong nor incoherent in being thankful for those people’s existence and questioning the way they were conceived, as stated by those people themselves, who are amongst the most fierce critics of TPR and related terminology, such as “donor-conceived” (Elizabeth Howard 2014). The procreative decision under analysis here absolutely precedes the existence of a being, for in the moment of that decision the person to be born is nothing more than an aspiration and does not exist not even as a cell. Now, as Parfit also shows through the non-identity problem, no harm can be done to someone who doesn’t exist at all. Likewise, nobody is harmed if people refrain from procreating for not being able to give the child to be born the company of both parents. As James Woodward writes “Considerations having to do with rights and fairness have a large role to play in population policies, even when the conditions of the non-identity problem are met” (Woodward 1986, p. 804).

## Love is not importable

If public discussion about TPR widely neglects the consequences of the underestimation of biological kinship ties, that is due, to a great extent, to the tendential association of the deconstructivist approach to the idea that “all you need is love”. Thanks to this successful case of political rhetoric, the discursive power of an attractive expression ends up serving the conviction that it is indifferent to a person to be loved by her parents or anybody else; as long as she is loved, it would not matter who loves her (Cholodenko 2010). In sum, within the idiosyncratic context of the current debate, affirming “all you need is love” is equivalent to assuming that love is something importable and the love of a person’s parents is irrelevant.

In support of this position, it is frequently invoked the undeniable dysfunctionality of many biological families, along with the equally undeniable robustness of affective ties between many non-biological parents and their children (Lansford et al. 2001; Hertz 2008; van den Dries et al. 2009; Biblarz and Stacey 2010). The problem of such arguing is two-fold.

First, it reflects a tendency to ignore the amount of evidence mentioned before about the disproportionate way in which people raised without one or both biological parents face diverse kinds of problems by comparison to those who benefit from the coexistence with both. In other words, to overemphasize the constructed side of affection means, in the first place, ignoring the well-grounded conclusion that, as a rule, the best for a person’s development in her infancy and adolescence is to be raised by her parents, as even adoptive people acknowledge (Rushbrooke 1998).

Secondly, its disregard for the importance of natural bonds between parents and children promotes the idea that “beyond insemination, fathering is fundamentally a social construction” (UN Department of Economic and Social Affairs - Division for Social Policy and Development 2011, 51). According to such perspective, fatherhood and motherhood are social roles whose connection with biology must be deconstructed, namely by defending that they can be performed by anybody else than a man and a woman or even the mother or the father. Transformed into a vehicle for the deconstructivist vision that there is no mother or father, but merely those who play the role of mother or father, the friendly statement “all you need is love” consists in a denial of the unique and unreplaceable place of natural parents in their children’s lives, thus emptying the biological connection between them of any existential meaning.

### “It is only a donor”, or the nominalist attempt to vanish fatherhood

A minimum degree of familiarity with the intellectual production related to “gender studies” shows that the vision of gender as a purely cultural construction has always predicted, in a more or less assumed way, profound implications for kinship (Wittig 1992; Butler 1998; Robaldo 2011). If we believe that the connection of femininity and masculinity to biological sex constrains individuals and is a source of oppression and discrimination, then, logically, the categories “paternity” and “maternity” are a primordial goal of deconstruction, given their conceptual link to biological sex. The mere existence of different notions to designate the parents – “father” and “mother” – would somehow prove the cultural influence of “biological determinism” and “heteronormativity” on social representations of the family, thereby repressing an alternative kinship paradigm (Anne Cadoret 2003; Levine 2008). Deconstruction can take place in practice by substituting the words “mother” and “father” for “parent A” and “parent B” (as well as “motherhood” and “fatherhood” for “parenthood”), for example (Radio Canada 2013; Gouvernement du Québec - Ministère de la Justice 2017). Another option is legitimizing reproductive biotechnology applications which perfectly illustrates that the disconnection between gender and biology may not always be just a one-way road: if it can serve to denounce the idea that women shall be mothers, it can also serve to promote the idea that mothers may not be women.

A crucial feature must be emphasised at this point: the deconstructivist vision not only affirms the indifference of having *a* father and *a* mother but also of having *the* father and *the* mother (and their respective love).

Kinship thus becomes a kind of clay, moldable according to the criteria of social engineering experiences within which biological parents are disposable, and children appear as exchangeable goods. Consequently, reasonings such as the following become normalised: since “affections are construed, ‘they are not in the blood’”, the newborn that a woman generates in her womb and delivers at birth is not necessarily her son. Since “affections are construed, ‘they are not in the blood’”, it is better to avoid breastfeeding and even separate the “surrogate” from the baby at birth, to prevent any kind of affective connection (Deonandan, Green, and Van Beinum 2012; Jennifer Lahl 2016; Allen 2018); since “affections are construed, ‘they are not in the blood’”, and “males have always been fathering anonymously and irresponsibly; why not put this otherwise noxious trait to good use?” (Rose 2009, p. 24).

These arguments are coherent derivations of the inspiring deconstructivism that feeds the disregard for the biogenetic affinity and denies beforehand the easily presumable aspiration of any person to be loved by her mother and father. By enabling a desire for children that involves the premeditated intention of depriving them in advance of their mother or father, the State legitimises these people’s discrimination. An apparently egalitarian vision results, therefore, in the inequality of children, as well as in other contradictions: “The whole procedure certainly runs contrary to our tradition that natural parents should ordinarily take moral, social and economic responsibility for their children” (Fisher, 1989, cit. in Rose 2009, p. 60). If governments aim to “help to embed a cultural norm that fathers should reach the birth of their child with an expectation that they have clear responsibility for their child” (Rose 2009, p. 62), then, how “is encouraging the intentional creation of a genetic child that one intends to have no responsibility for, a healthy paternal (or maternal) culture for the government to promote” (Rose 2009, p. 60)? How can the premeditated and collectively organised disruption between biological siring and responsibility be conciliated with “the normative recognition and expectation that unless there are circumstances that jeopardise the child’s wellbeing, natural parents should be supported to take this responsibility seriously” (Rose 2009, p. 60)? Is not that disruption somehow anachronic in a time full of “appeals to increase fathers’ interaction and input into child-rearing, indeed to raise the role of the father” (Rose 2009, p. 61)? Judge John Smoot exposes the same argument by recalling Margaret Mead’s idea that “the supreme task of any society is to teach its men to be good fathers” (Smoot 2013). As Joanna Rose concludes, “the absence of one’s father tends only to be painted in a positive light, or indeed in an irrelevant light if it is pragmatically necessary to do so as part of the justification for the intentional creation of such absence” (Rose 2009, p. 69). Rupert Rushbrooke, an adopted son himself, resumes the problem:

as new technologies have been introduced [and continue to be introduced] that are also predicated on the notion that blood relationships are trivial, the number of people damaged by this industry will be even greater, and the effects may take the form of social, as much as individual, problems> (cf Rose 2009, p. 211).

### Intimate, unrepeatable, and inexorably personal

Expressions such as “affections are construed; they are not in the blood”, “donor children” or “all you need is love” reflect the process of “mediation of meanings” that Roger Silverstone and Nomi Sunderland (Silverstone 1999; Sunderland 2004, 2008) describe as a mechanism of language shifting and adjustment. This mechanism is a good prism to introduce the “seemingly invisible process whereby (…) biotechnology is engaging in and affecting the social and biological functions of reproduction, kinship and identity” (Rose 2009, p. 17). The mediation of meanings “narrows down” the inclusion of specific analyses and “broadens the exclusion” of others, thus promoting, in the specific case of sperm donation, an “unbalanced and ill-considered picture” in which many “donor-conceived” people (and respective problems) “are made less ‘visible’ and hence (…) not given their proper weighting or consideration in the usual ethical analysis of this practice” (Rose 2009, p. 18). As defended by Brenda Almond there is a genetic and cultural heritage whose relational substance tends to be devalued:

If gametes are regarded as being no more than raw material for the medical manufacture of children, a whole dimension of human reproduction is lost – in particular, the network of kinship relations that provides the key to an understanding of society’s culture and practices. (Almond 1998, p. 142).

The “alienation of fatherhood”, or the denial of the personal affinity of the biogenetic connection between “donor father” and “donor-conceived” children, exemplifies how the mediation of meanings in the context of sperm donation promotes “a destructive notion of paternity that is decisional, contractual, alienable, instrumental, fractured, and even commercial” (Rose 2009, p. 18). The idea that people conceived via sperm banks have no father, but a “donor” plays a central role in this context. It perfectly reflects the process of mediation of meanings, namely through the assimilation of the assertion that paternity is a purely social construction that can be eliminated or transferred by decree, as if the juridical designation of biological fathers as donors amounted to a sort of magic trick that effectively transforms fathers into donors (and harms none). The Bioethics Committee of the Council of Europe addressed this issue in a recent study:

[third party reproduction] has generated considerable concern about the rights of children to information, not only regarding identity and to know one’s parents, but in part concerning who is legally registered by law as the child’s parents (…) Some of these problems are created not by the technology itself but by national laws – for example, those that shield donors from registration as parents or only recognize parents based on marital or adoptive status rather than biology. (Zillén, Garland, and Slokenberga 2017, 24–25).

As Joanna Rose affirms, “The offspring’s kinship and identity are then framed through an experimental postmodernist notion, presenting them as social rather than innate constructs” (Rose 2009; ii).

The physical and psychological similarities with one’s parents are perhaps the most immediate invitation to understand a connection that is inexorably biographical and not merely genetic. As Vardit Ravitsky defends, when the right to know biological origins is denied, the freedom of people conceived via TPR to choose what meaning to attribute to critical components of their identity is automatically violated. By being forced to consider their origins irrelevant, they are forbidden to see their own story as an essential dimension of their life and personhood (Ravitsky 2012, 36–37). Vellemann’s position is similar: knowing biological parents is due to every person and this means not just having information about one’s relatives but also having *acquaintance* with them. Only through that acquaintance, we can sense the “intuitive and unanalyzable resemblances” we have to our biological relatives. That *intuitive and unanalyzable resemblance* structures a sort of self-knowledge and a narrative that gives meaning to our actions (Velleman 2005, p. 368).

Given the similarity of situations regarding the absence of at least one of the parents, it seems reasonable to conclude that people conceived through sperm banks face identical problems to those of adoptive children throughout their childhood and youth development, namely problems associated with “genealogical bewilderment*”*. This is admitted by researchers who are adopted people themselves (Rushbrooke 2001, 33–34). Indeed, as ironic as it may be, as demonstrated by multiple testimonies of “donor-conceived” and similar experiences, the concerns that, by introspection or comparison, affect the experience of orphans, abandoned, or adopted, may become more unbearable when the parent’s absence is surmountable than when it is insurmountable. The biological connection is expressed in daily interaction and socialization, namely in the importance that might have for a person’s self-representation to know parents, siblings, cousins or grandparents, the story about her origins, the physical and personality traits she inherits, etc. (Callahan 1992; Martin 2017). First-hand testimonials show that a considerable part of these people struggle with family, psychological, emotional and affective problems due to the tension over their origin and identity, experiencing feelings of loss, confusion, sadness, anguish or rejection due to not being able to live or even know his father and paternal family (Clark 2006; Luden 2011; Faust 2015). Those feelings operate at a somewhat unfathomable level, making it difficult to assess the profound impact of losses and disadvantages that belong mainly in the subconscious domain (Pruett 2001; Comité Consultatif Nationale d’Ethique pour les Sciences de la Vie et de la Santé 2009, p. 15). As stated in a public policy guideline document from the British Government, “Father-child relationships, be they positive, negative or lacking, have profound and wide-ranging impacts on children that last a lifetime” (Rose 2009, p. 62). We talk of relational losses - or voids - and not only of information.

At this point, it is pertinent to point out a typical feature of the “absorption” dynamic that Sunderland also frames within the referred process of mediation of meanings (Sunderland 2004). It consists of many “donor children”’s tendency to sacrifice their identity concerns for fear of hurting their parents. On the one hand, there is pressure for resignation: everybody, especially “donor-conceived” individuals, is pressured to conform to the positive vision of sperm donation that parents, politicians, or the fertility industry offer. On the other hand, there is also a demand to conform to the pressure: everyone, especially “donor-conceived” individuals, is pressured to give thanks for the birth of these people, as if questioning the method of their conception (and the State’s stance on it) meant questioning the value of their existence. This dynamic helps to explain why the criterion for defining the child’s best interest shall not be the assumption that “children are okay”, but rather the principle of precaution, as Rupert Rushbrooke defends:

I think there is a serious problem with the industry’s terminology…[the term donations implies that]the processes concerned are just about sperm or eggs, and are no different to donating organs, money or blood. It is implied that the identity of the natural father or mother is not important. I would rather assume that they are important unless it is proved otherwise (cf Rose 2009, p. 15)

As Brenda Almond emphasises, what is at stake is not only the lack of the father but of half the family: “The extended network of kinship made up of grandparents, brothers, cousins, uncles and others — a network of connections that constitutes the social space within which a person also finds his original identity” (Almond 1998, p. 142). Alexina McWhinnie, an author with many years of research dedicated to the study of people adopted and conceived by TPR, addresses the same point while pointing out the mediatic influence of studies based either on donors’ and sperm bank users’ “self-interview questionnaires”, compared to the less influential studies based on systemic analyses of children’s social and interpersonal relationships (McWhinnie 1998).

The conversion of TPR from an extreme remedy to medical problems into a normal alternative method of procreation (available to everybody), is only possible thanks to the disregard for the biological and social continuity, or nexus, that already Aristotle considered constitutive of fatherhood (and motherhood), as the very etymology of the word “filiation” suggests — from the Greek “*philia*”: friendship^4^. Trivializing conception through sperm donation distorts kinship by intentionally disregarding its dual structure. Instead of benefiting from both parents’ company and love, most “donor-conceived” children are only generated as long as their father relinquishes the responsibility to raise them and remains absent or even anonymous. The conception of people deliberately deprived of their natural father becomes, in brief, an instituted and collectively organized practice. Such a situation reveals an immediate contradiction since it validates some people’s desire for biological connection at the expense of acknowledging the legitimacy of that same desire in others. Instead of being understood as a subject, the child appears more as an object, since her existence results not from the parents’ convergent will to create and raise her, but precisely because of their divergent intentions in that regard.

It is undeniable that biological parents’ physical presence is not synonymous with affective presence, and in extreme situations, it may even be beneficial to separate them from their children. It is also true that people can develop without problems thanks to other people’s love. However, as Melissa Moschella argues, it would be a mistake to believe that single mothers, adoptive parents, stepparents, or even other family members can truly replace one’s mother and father, even when they are unknown (Moschella 2017). The parent’s right to raise their offspring derives from their duty to love, a duty associated with the unrepeatable place they occupy in their children’s lives and which makes their love inexorably personal and non-transferable:

The relationship between children and their biological parents is intimate, permanent, and identity-constituting. It defines the biological aspect of the child’s identity—for if the child had different biological parents, he would not be the same person; indeed, he would not exist at all. Children do not miss being loved by those with whom they have no intimate relationship; the unique, irreplaceable intimacy of the parent-child relationship manifests itself in the fact that a child can miss the specific love and care of an absent biological parent, even when she is well-loved by (say) adoptive parents. (Moschella 2015).

[T]he parent-child biological bond really does matter in itself because there is at least one unique and important benefit that biological parents—and only biological parents — can provide for their children: their parental love. (…) Certainly, when biological parents cannot or will not raise their children themselves, others can generously take on that task and do an excellent job. (…) But their love cannot replace the absent love of the child’s biological parents any more than the love of another man or woman can replace the love of an absent or deceased spouse. Biological parents, simply by virtue of their biological (genetic) connection to their children, have an intimate and personal relationship with those children that makes their love irreplaceable. The absence of their love is not like the absence of a stranger’s love, because, even if they have never actually met, biological parents are not strangers to their children. (Moschella 2014).

The biogenetic connection makes the relationship between parents and children truly intimate, thus, the void left by parents in children’s life, even if they never knew each other, can only seem strange if biological parents are seen as no more than mere strangers to their children. Elizabeth Marquardt reinforces Melissa Moschella’s vision and emphasises the insensitivity of the phrase “My daddy’s name is Donor”, which an association adopted as a slogan in favour of universal access to sperm banks (Marquardt, Glenn, and Clark 2010). As Marquardt shows, it is paradigmatic of the disseminated ignorance about the demonstrated harms of the father’s absence.

### Complementary clarifications

It must be highlighted that the ethical problem discussed here does not consist in the decision to have a child despite the impossibility of giving her her father’s company due to any unexpected and unwanted cause, like death, separation, negligence, or paternal incompetence, for example. An ongoing connection with biological parents is so significant that it is wrong to deprive someone of it *without any compelling reason*. The point here is the decision to have a child with the intention of depriving her of her biological father for no other reason than her parents will. This is exactly what happens in most cases of access to sperm donation: people undertake to create a child that the biological father has no intention to raise and, on many occasions, does not even want to know. Can this be seen as a compelling reason equivalent to those contingent and unfortunate facts previously mentioned? In our view, it cannot, independent of whether the mother believes that she can provide everything that the child needs. The problems related to the father’s absence in critical ages (especially if he remains anonymous), don’t depend on the intended mother’s goodwill. Notwithstanding her love and dedication, which may well be unquestionable, her daughter/son may still lack her father’s love and suffer because of it in different ways, as corroborated by multidisciplinary scientific research.

This position shall not be confused with the anti-natalist position according to which all acts of procreation are wrong since bringing somebody into the world inevitably causes suffering. To fully justify this stance, it is important to emphasize the action’s intentionality, namely the desire to neither know nor be known by one’s biological son or daughter.

A good way to better illustrate the ethical problems underlying donor insemination is to compare TPR with adoption. Giving a child to adoption always entails depriving children of their biological parents, but this alone doesn’t make adoption morally wrong at all, because adoption aims precisely at compensating already existing children who, for multiple undesirable reasons, cannot grow up with their biological parents. It is not grounded on the assumption that natural bonds are irrelevant and parents should be free to disappear from their children’s lives or to handle them to others. This is exactly why it is forbidden to establish an adoption contract between biological and adoptive parents upfront, i.e., before birth. Such a pre-birth agreement between intended couples of biological and adoptive parents would amount to ordering people, i.e., to its objectification. Here lies, indeed, a critical difference: adoption is a regulated process in which the State intervenes to a protect child’s rights, in full conformity with the noble and structuring *leitmotiv* of adoption — giving a family to a child and not a child to a family —, whereas conception by donation is a highly deregulated market process where the rights *of* the child are obliterated in name of a supposed right *to* the child. In adoption, intended parents are submitted to long and demanding selection processes, a feature due to the need to check, among other things (socio-economical conditions, etc.), whether there are no pre-existing agreements of “people ordering”. This feature perfectly reflects the primacy of the principle of the child’s best interest in adoption, but it is neglected in the context of sperm donation (Brewaeys 1996; Kirkman 2003; Morrice 2007).

Both adopted and “donor-conceived” people deal with relational and existential losses, but if similarities between them already show why the principle of precaution must be a vital criterion for defining the paramount rule of the child’s best interest, the differences demonstrate it in a complementary way. It can even be misleading to compare both situations because similarities can obscure feelings that might indeed be more traumatic for “donor-conceived” than for adopted people. In the case of adoption, child abandonment is often involuntary and painful; in gamete donation, it is intentional and viewed with indifference (“affections are construed; they are not in the blood” and “all you need is *somebody’s* love). In adoption, parents often give their children away because they cannot raise them; in “donor conception”, children are delivered to others by their parents because only the mother wants to raise them and previously agrees with their father (directly or through the mediation of a clinic) his respective absence or even disappearance. Hence, it shall not be surprising that in addition to shared feelings of loss, emptiness and lack of sense of identity and narrative, “donor-conceived” people also experience abandonment and rejection, which are due precisely to the acknowledgement that, far from wanting to raise them, their biological father had no intention but to relinquish that role, transferring it in someone else and, in most cases, to abandon them from the beginning. In sum, adoption doesn’t owe its well-deserved statute of a good and just institution to the denial of biological parents’ importance, but precisely to the fact of presupposing it: adoption is good because it compensates the natural parents’ absence, instead of devaluing and promoting it.

In the face of all the responses already given to possible objections, A main argument arises in favour of a stronger protection for the existential density of biological affinity within the context of human reproduction in general, and of TPR in particular: the idea that while some causes of suffering are accidental, like poverty, others are deliberate and controllable, like the premeditate exclusion of a father from his child’s day-a-day life, an exclusion not due to contingent circumstances, but to the adults’ will. Poverty is also a good example to invoke in this context, given its status of an undesirable reality that nobody directly controls even though everybody hopes to eventually overcome it. In order words, we know that every poor person wants to escape from poverty and may ultimately do it. On the contrary, in most TPR cases through sperm banks, there is no intention to correct the very concrete cause of suffering. In fact, the tendency is to ignore it as if it was innocuous or irrelevant. This deliberate intention to erase the father shall be highlighted once more, for we are not dealing with accidental contingency or hazardous possibilities here.

In conclusion, it is not exactly the lack of connection with the biological parents that makes it justifiable to regulate TPR, namely by ending the normalization of sperm bank access. It is the decision to generate people who will be denied the company of their fathers (or mothers) based on the presumption that that company is irrelevant — irrelevant to the point of being denied simply because of the parents’ will, not by unpleasant (and so often unpredictable) life circumstances that generally justify the separation between biological parents and children (economic fragility, carelessness, divorce or harmful couple relationships, death, etc.)

## Conclusion

The knowledge resulting from comparative research about biological and non-biological families strongly confirms the disadvantages of paternal and maternal absence in child and adolescent development. This fact explains why that absence is not regarded as something to be promoted. On the contrary, what has been recognized as something in conformity with the paramount principle of the child’s best interest is a person’s right to be raised and loved by her parents. Logic and empirical evidence show that women unrelated to a man are the most interested population interested in TPR through “donor insemination”, hence biological father’s absence tends to become a consequence of the normalized access to sperm donation, i.e., as if it was an alternative way of reproduction. This poses an immediate challenge to the child’s best interest, whether the “third parties” are known for women who use sperm banks or not because they are never “third parties” to those who will be born.

People share a familial history with their parents whose omission harms the daily exercise of presentation and representation to oneself and others. They owe them their existence, their ancestry, some of their personality traits, and physical and psychological characteristics. To undervalue crucial aspects of the well-being and personality development of people conceived through donor insemination, such as knowing and feeding their origins’ (hi)story or their personality traits, is to underestimate a heritage that is one of the most primordial inductors of a person’s sense of belonging. That heritage is crucial for an individual to recognize essential coordinates of personal identity and self-knowledge. To ignore the personal dimension of the biological affinity is to favour impersonality; it is to sabotage both the interactive and introspective dimension of the subject or, in other words, a person’s ability to present and represent herself; it is to promote the individual’s atomization, his disintegration and depersonalization, his strangeness before others and before himself. In a few words, we need to know where we come from to know who we are.

This perspective is framed in an ontology that emphasizes the relational entity that every human being is in its essence, in its origin, and not only in its exterior or as a social being who relates with others. Alerting nowadays tendency to neglect personal and existential dimensions that gravitate around TPR, is in harmony with the scientific and historical path about the importance of biological connection for the construction of individual personality. It also respects a crucial idea: we can properly say that we know somebody when, besides knowing *who* she is, we also know *how* she is, which necessarily presupposes a minimum of conviviality and coexistence. That is even more justified if those to be known are the two people directly responsible for one’s life. This argument is fully reflected by the etymological origin of the word “filiation” (“*philia*”).

It is a profound irony that, in the context of the present discussion, the statement “all you need is love” is more influential the more it ignores the importance of a father’s love and presence in the lives of people conceived via “donor insemination”, most of whom grow up in fatherless homes. This ignorance explains, to a great extent, the process through which normalizing the father’s absence ends up being seen as something compatible with the paramount principle of the child’s best interest. To fully understand this ironic phenomenon it is necessary to consider the synchronic influence of conceptual and discursive mechanisms that promote the vision of a person’s biogenetic heritage as something irrelevant and neglect the biographical meaning of biological affinity. It is indeed paradoxical that the statement “all you need is love” may be now used to demand the normalisation of a practice that consists of generating fatherless people beforehand. The truth is that the expression’s projection increases in direct proportion with the contempt for the personal and intransmissible nature of the father’s (or mother’s) love since not even an absent father can ever be considered a stranger to his child.

As scientific evidence rejects the thesis that TPR is harmless for children, consistent proof shall be demanded from those who want to accelerate such disruptive interventions, not from those who want to stop it. As argued by Elizabeth Marquardt, her “and ample other work is showing that indeed the resulting persons [from donor conception] can be harmed by this practice [TPR]”, but in any case, the burden of proof lies with who defends the institutionalisation of policies and practices that operate a complete revolution in how society sees kinship, specifically by deliberately depriving children of their father or mother at the very moment of the procreative decision (Elizabeth Marquardt 2010).

The claim that TRTs are not problematic for children owes a lot to the promoted or self-imposed silence of adult “donor children” who do not confirm that view. Some questions arise in this context: what is the limit for parent-child separation once it is assumed that biological affinity is insignificant? If we believe that the biological connection between a person and her mother or father has no significance because “all you need is love” and they are nothing more than a “surrogate” or a “donor”, will it be reasonable, or instead somewhat naive, to expect that empirical evidence on personal problems might ever come to be considered strong enough to slow down or even question TPR?

Heterologous reproductive biotechnology rehabilitates in a renewed way timeless teachings on the value of parent-child bonds and the importance for human beings to be welcomed and loved by their natural mother and father. One of those teachings is that we cannot know who we are if we do not know where we come from. The existing scientific evidence corroborates this teaching and invalidates the conviction that family affinity is purely constructed and re-doable without loss or harm. Even though the parent-child relationship might not be cultivated, their bond is intimate, permanent, and constitutive of identity, thus making a father’s/mother’s love something irreplaceable. The inalienable and inexorably personal character of that relationship explains why a person may suffer for not having her mother/father’s love even when she is loved by adoptive parents or grandparents, for example.

It is questionable that the conversion of TPR into an alternative means of reproduction can be justified in the name of equality and the fight against discrimination. Indeed, that step ignores the inequality of people treated at birth as if they never had a father. This inequality results from contradictions that the following question resumes: if the relevance of biological ties is acknowledged for the life and personality development of adults who resort to sperm banks, how can it be assumed that those same ties are irrelevant for the life and the personal development of the children? How can this incoherence be sustained, when it means normalizing the generation of someone who will be voluntarily denied the right to have her father’s presence and love? Other contradictions follow from what was said: nowadays, public policies reflect an increasing concern in freeing parents to intervene in their children’s lives, censuring conduct that systematically delegates parental responsibilities to other people. If the paternal absence is alarming and censurable when it is frequent, how can it be irrelevant when it is permanent and lifelong?

The formulation of a supposed right *to* a child suggests an unconditionality that would imply the annulment of the right *of* a child to be loved and raised by her parents. It would normalize a rupture that the rule of law has decided to compensate (through adoption, for example) precisely for considering it undesirable and not acceptable in principle. Hence, the difference between the right *to a* child and the right *of a* child is not a mere terminological detail. On the contrary, it accurately points out a necessary and fundamental ontological distinction: every person is always a relational fruit — *someone* — and never an object or even a project of a purely individual will — *something*. How can then the legitimate and understandable desire for reproduction be met and collectively organized unconditionally, specifically when the decision to generate descendants denies beforehand another person’s right to know the ancestry?

Sylviane Agacinsky and Jacques Testart defend that the trivialisation of sperm banks is a step towards the objectification of both children and men, in which the latter become a sort of sperm reservoirs and are allowed to disregard any responsibility for their offspring (Sylviane Agacinski 2013; Jacques Testart 2017). In this sense, it could be said that far from being anti-naturalistic progress, that step means an anachronic return to a time when it was more or less usual and culturally accepted for men to imitate most male mammals’ behaviour regarding offspring (Rohner 1998).

This article shows that biological connection is indeed so important that societies have the moral duty to respect it and procreative decisions are unjustified whenever based on a voluntary contempt for the importance of that connection. In the context of that effort, which tries to conciliate in a satisfactory way existing scientific evidence and philosophical reasoning, we emphasize the ethical importance of the question of intentionality, i.e., the fact that there is a premeditate intention to relinquish the father’s responsibility to raise the children to whom he gave life for no other reason than an individual desire to procreate (which can hardly be seen as a proper compelling reason). That intentionality feature distinguishes donor insemination decisions from those accidental, unpredictable, or undesirable cases where the absence of biological father is unavoidable or even justified for the child’s best interest (death; unhealthy relationships that harm children’s development and well-being, extreme poverty, etc.).

In conclusion, procreative acts resulting from decisions that intentionally and deliberately violate the prospective people’s right to their father’s care are unjustified in the same exact way that non-consented procreative acts are unjustified even if they result in a new person. If this person’s dignity is certainly unquestionable, the argument according to which we must accept the referred acts because if it was not for them that person wouldn’t exist, is not. None of the referred procreative acts (and respective violation of basic rights) shall be justified and allowed just because some (or even many) people owe their lives to those acts and are happy or thankful for living. To put it in another way, if some couples decided to conceive children with the intended purpose of handling them to others (thus violating basic rights, including the right to be loved by their parents), should society accept that decision on the argument that if it was not for it, those children would never exist? Would the ethical evaluation of such a decision change if those children assumed to be happy and thankful for their lives? If we recognize that no one has the right to intentionally deprive children from their father’s love and care (as scientific evidence also suggests we shall do), the decision to violate that right remains unjustified even if it brings a new person into existence. Although the outcome of a wrong act may ultimately be positive, that doesn’t turn that act into a good act nor justifies it.

The contradictions, conceptual inconsistencies and ethical problems described here are not solved through the assimilation by decree of the conviction that it is irrelevant to know and be raised by biological parents, or that some people have donors instead of fathers or mothers. It is ironic that approaches of this type are considered realistic and “inclusive”, whereas the defense of the right to be raised by one’s mother and father is associated with “hate speech” and intolerance. Complementary analyses of the problem could explore more deeply this and other mediatic fallacies.

## Data Availability

The author confirms that all data analysed during this study is included in this published article and can be accessed in the references.

## Footnotes

1 In its rule 225/2018, that declared the inconstitucionality of some clauses of the Portuguese ART law of 2016, namely that of the anonimity of sperm or egg donnors, the Portuguese Constitutional Court endorsed the position of the Portuguese National Council for Ethics and Life Sciences (CNECV) against “a paradigm shift on the use of ART”, which “puts the focus of all protection exclusively on the woman, thus neglecting that group of rights which constitutes the most important value to safeguard, and towards which the State has a special duty of protection: the rights of the child. If the Constitution protects the right to form a family, it also protects the right of each person to know her own identity — including the genetical —, and between both, it is the former that has to yield, not the latter.” (p. 1886).

2 Physical and mental health; emotional well-being; social insertion; cognitive performance; personal identity; self-confidence, etc.

3 As Joanna Rose points out, it seems incoherent to hold such view for someone who defends abortion, as it was the case of Emily Jackson. Indeed, if one affirms a right to life that applies even to those who don’t exist at all and are only projects of somebody else’s will, how can then deny the right to life of a being that already exists as fetus, even if we don’t recognize it as a person?

4 Aristotle, *Politics*, Book II, § 4; Aristotle, *Nicomachean Ethics*, Book VIII, § 12.
